# Engineering of L-amino acid deaminases for the production of α-keto acids from L-amino acids

**DOI:** 10.1080/21655979.2019.1595990

**Published:** 2019-03-27

**Authors:** Project Nshimiyimana, Long Liu, Guocheng Du

**Affiliations:** a Key Laboratory of Carbohydrate Chemistry and Biotechnology, Ministry of Education, Jiangnan University, Wuxi, China; b Key Laboratory of Industrial Biotechnology, Ministry of Education, Jiangnan University, Wuxi, China

**Keywords:** α-keto acids, directed evolution, L-amino acid deaminase, error-prone PCR, site-saturation mutagenesis

## Abstract

α-keto acids are organic compounds that contain an acid group and a ketone group. L-amino acid deaminases are enzymes that catalyze the oxidative deamination of amino acids for the formation of their corresponding α-keto acids and ammonia. α-keto acids are synthesized industrially via chemical processes that are costly and use harsh chemicals. The use of the directed evolution technique, followed by the screening and selection of desirable variants, to evolve enzymes has proven to be an effective way to engineer enzymes with improved performance. This review presents recent studies in which the directed evolution technique was used to evolve enzymes, with an emphasis on L-amino acid deaminases for the whole-cell biocatalysts production of α-keto acids from their corresponding L-amino acids. We discuss and highlight recent cases where the engineered L-amino acid deaminases resulted in an improved production yield of phenylpyruvic acid, α-ketoisocaproate, α-ketoisovaleric acid, α-ketoglutaric acid, α-keto-γ-methylthiobutyric acid, and pyruvate.

## Introduction

Keto acids or oxoacids, are organic compounds that contain a carboxylic acid group and a ketone group. Among the three types of keto acids (α, β, and γ), α-keto acids have received the most attention, because they are involved in the tricarboxylic acid cycle and glycolysis [1]. The α-keto acids are the primary products of amino acid biosynthesis. They are also of interest for intermediate chemical syntheses as model substrates for enzymes and for the development of enzyme inhibitors. Patients with renal failure can avoid the risk of inadequate amino acid intake, due to their protein-restricted diets, by taking a deaminated α-keto analog, which has been proven to reduce the risk of nitrogenemia [1–5].

**Table 1.** Comparison of α-ketoacids production using wild type and evolved L-AAD.

Produced ⍺-keto acid	Source of L-AAD	Whole-cell biocatalyst	Substrate	Wild type LAAD titer	Evolved L-AAD titer	References
Phenylpyruvic acid	L-AAD from *Proteus mirabilis*	*Escherischia coli* BL21(DE3)	L-phenylalanine	3.3 g/l with 82.2% substrate conversion rate	10 g/l with 100% substrate conversion rate	[34,35]
α-ketoisocaproate	L-AAD from *Proteus vulgaris*	*Rhodococcus opacus* DSM 43,250	L-Leucine	1,264 g/l	86,55 g/l with 94.25% conversion rate	[39–41]
	L-AAD from *Proteus vulgaris*	*Escherischia coli* BL21(DE3)	L-Leucine	69.1 g/l with 50% substrate conversion rate		
α-ketoglutaric acid	L-AAD from *Proteus mirabilis*	*Bacillus subtilis* 168	L-Glutamic	12.79 g/l	89.11 g/l when Ep-PCR and gene shuffling are integrated	[46–48]
α-ketoisovaleric acid	L-AAD from *Proteus myxofaciens*	*Escherischia coli* BL21(DE3)	L-Valine	2.014 g/l	8.197 g/l	[50]
α-keto-γ-methylthiobutyric acid	L-AAD from Proteus vulgaris	*Escherischia coli* BL21(DE3)	L-Methionine	2.4 g/l with 71.2% bioconversion rate	increased molecular conversion rate up to 91.4%	[54]
Pyruvate	L-AAD from *Proteus mirabilis*	*Escherischia coli* BL21(DE3)	L-alanine	5.38 g/l	14.54 g/l	[59]

**Figure 1.**
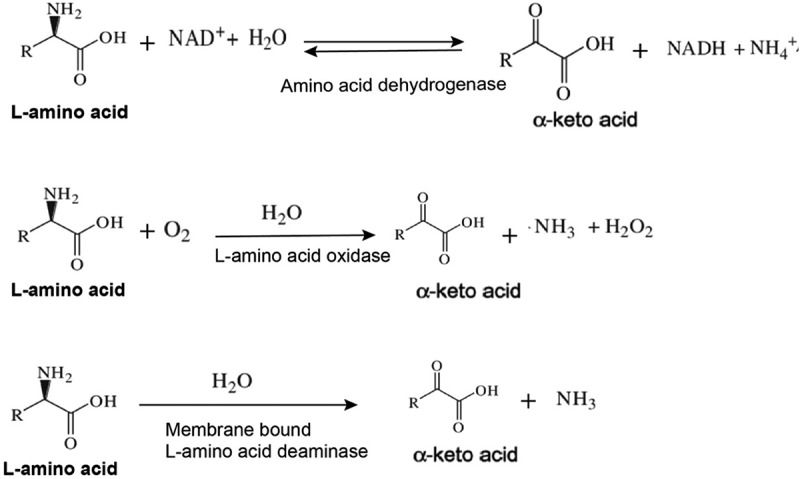
Mechanism of different enzymes catalyzing the amino acids to α-keto acids.

L-amino acid deaminases (L-AADs) and L-amino acid oxidases (L-AAOs) are flavin-containing enzy-mes that catalyze the oxidative deamination of amino acids for the formation of their corresponding α-keto acids and ammonia (Figure 1). These enzymes are commonly expressed in snake venom, insects, fungi, fishes, and some bacterial species, but primarily the *Proteus* species. Membrane-bound L-AADs from *Proteus* species have been shown to perform well in heterologous expression systems for the production of α-keto acids using whole-cell biocatalysts. L-AADs can be used to develop novel biocatalytic processes, including the production of optically pure D-amino acids and their corresponding α-keto acids. L-AAD variants are preferred for use in whole-cell biocatalysis over L-AAOs, because they do not produce H_2_O_2_ as a byproduct within the cell [6–14].

α-keto acids are synthesized industrially via five chemical processes: the hydrolysis of acyl cyanide, hydrolysis of oxime ester, hydrolysis of ethyl esters of oxalo acids, hydrolysis of the addition product of Grignard reagents with diethyloxamates and the hydrolysis of azlactones. Some of these chemical methods use harsh reaction conditions and chemicals such as cyanides and needs multiple purification steps which results in plenty of waste [1,15]. Another way of producing α-keto acids is through the enzymatic method. In this method, the D or L-amino acid is oxidized by the action of amino acid oxidase to the corresponding α-keto acid, hydrogen peroxide and ammonia. The hydrogen peroxide is rapidly removed by the action of catalase to prevent the oxidative decarboxylation of α-keto acid by hydrogen peroxidase, which otherwise could produce carboxylic acid containing one less carbon than the parent amino acid [1]. L-amino acid can also be produced by the oxidization of L-amino acid with dialyzed snake venom (which contains L-amino acid oxidase) in the presence of excess crystalline beef heart catalase [16]. However, the production of α-keto acids via enzymatic transformation has also some disadvantages; the accumulation of α-keto acids causes a lower cell growth rate and requires costly supplementations [17].

**Figure 2.**
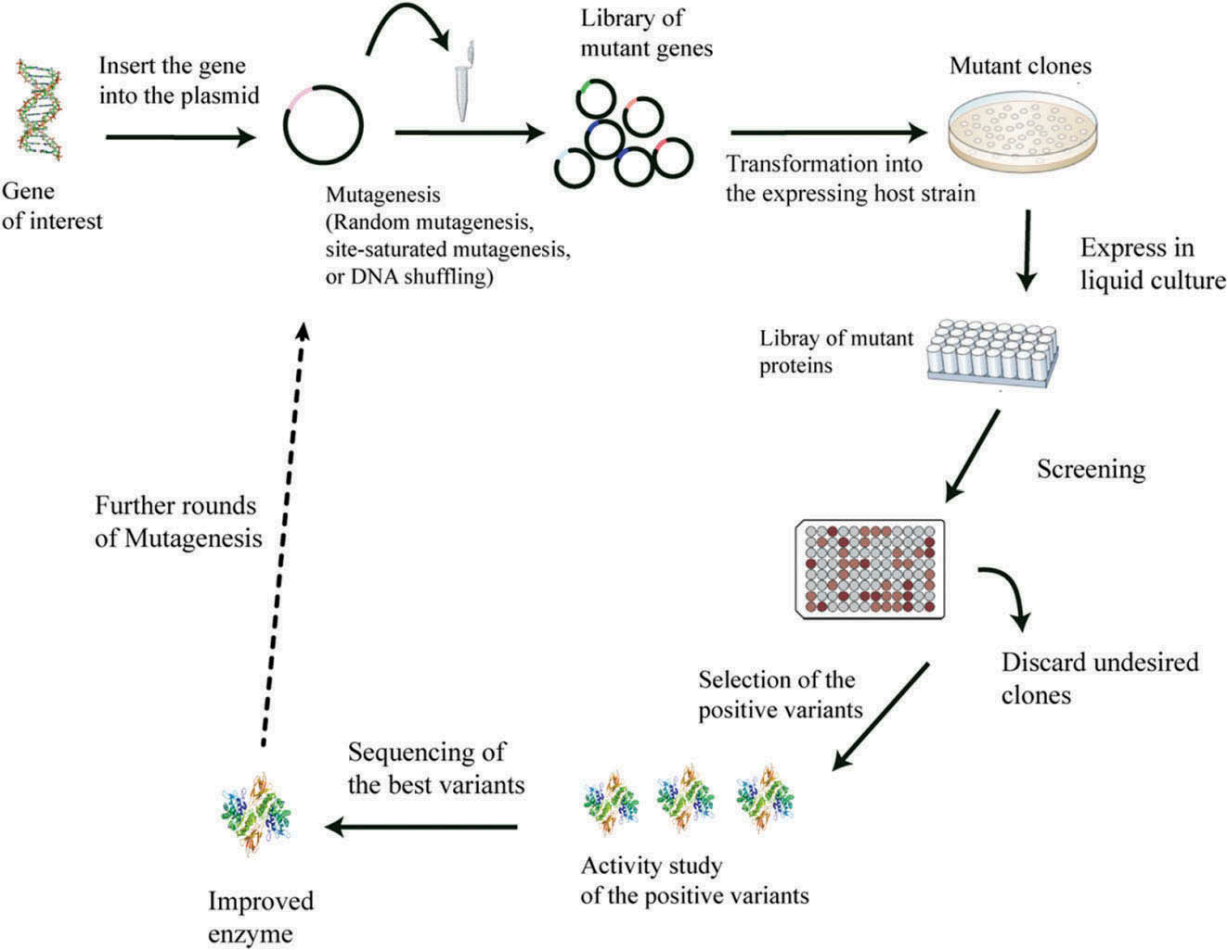
Schematic illustration of directed evolution method.

**Figure 3.**
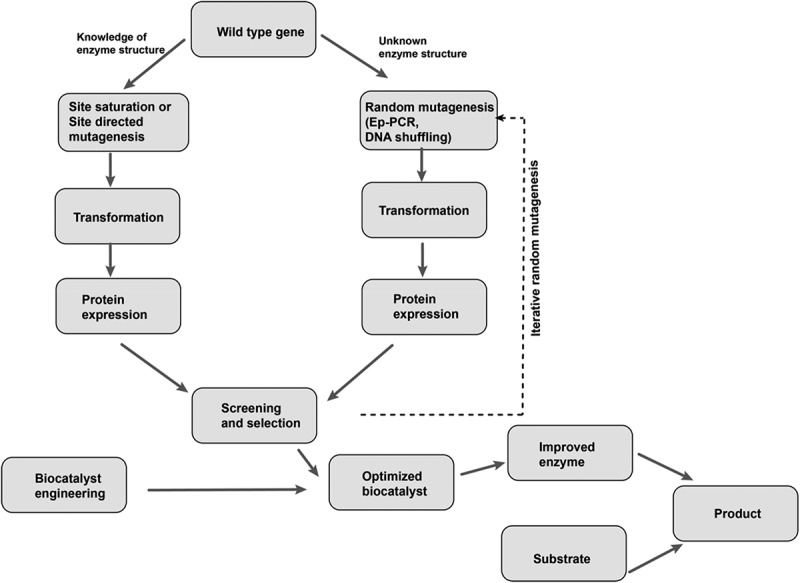
Diagram depicting strategies used for directed evolution in biocatalysis.

Directed evolution is defined as an iterative round of random or targeted mutagenesis, followed by the screening and selection of desirable variants [18]. This technique mimics Darwinian evolution (ie the survival of the fittest in nature) in the laboratory test tube, and it has proven to be an effective way to engineer enzymes with improved performance or new reaction specificities [19–23]. In the laboratory, the spontaneous mutation rate is generally insufficient to reach the number of desired gene variants. Therefore, genetic diversification techniques, including directed evolution, are useful for the generation of libraries of variants and novel biocatalysts [24]. Directed evolution is achieved through a library creation technique that involves either the randomization of one codon of a gene (site-saturation mutagenesis) or the introduction of mutation, with a relatively even mutational spectrum, via error-prone PCR (Figure 2). Some studies have reported that saturation mutagenesis yields better variants than error-prone PCR, but this technique relies heavily on knowledge of the molecular structure of the functional regions of the enzyme [25,26]. Directed evolution has made protein engineering readily accessible to scientists. Engineers that wish to generate enzymes with new reaction specificities or to improve the performance of existing enzymes (eg those with poor stability, low reaction rates, product inhibition, limited substrate conversion, etc.) have embraced this technique (Figure 3) [20,27]. In this review, we have selected recent applications of the directed evolution of amino acid deaminases for the production of α-keto acids. We discuss more about α-keto acids in general, whole-cell biocatalysis and more importantly, the directed evolution of amino acid deaminases for the improved production of α-keto acids (Table 1). We summarize the recent progress in the biological production of some α-keto acids (phenylpyruvic acid, α-ketoisocaproate, α-ketoisovaleric acid, α-ketoglutaric acid, α-keto-γ-methylthiobutyric acid, and pyruvate) that so far have been studied.

## Recent applications of directed evolution for the improved production of α-keto acids

### Phenylpyruvic acid

Phenylpyruvic acid (PPA) is an α-keto acid derived from phenylalanine. It is generally used in the pharmaceutical, food, and chemical industries [17,28,29]. PPA can be used as the starting material for the production of D-phenylalanine [30]. In the laboratory, when using *Escherichia coli* cells expressing D-2-hydroxyisocaproate dehydrogenase from *Lactobacillus delbrueckii* and formate dehydrogenase from *Mycobacterium vaccae* N10, the PPA can serve as the substrate for the production of phenyllactic acid [29,31].

Commercially, PPA is produced by several chemical methods, including the use of high pressure, from benzyl chloride and carbonic oxide, using the octacarbonyl form of cobalt as the catalyst [32]. The production of PPA via the chemical method is costly and produce many toxic byproduct [33]. Thus, its production through the use of a recombinant *Escherischiax coli* whole-cell biocatalyst has the potential to be a good solution.

Hou et al. (2015) produced phenylpyruvic acid from L-phenylalanine via a one-step biotransformation using the L-AAD from *Proteus mirabilis* KCTC 2566 cloned and expressed in *E. coli* BL21 (DE3). The maximal production was 3.3 g/L with a mass conversion rate of 82.5% [34]. However, the PPA titer was low due to the degradation of PPA and the low substrate specificity of the L-AAD. To resolve that problem, metabolic engineering of the L-phenylalanine degradation pathway in *E. coli* BL21(DE3) (i.e. Three aminotransferase genes; *tyrB, aspC*, and *ilvE* were knocked out from the *E. coli* BL21(DE3) to block the PPA degradation) and the directed evolution of the L-AAD were performed in an attempt to improve the PPA titer. After the knock out, the L-AAD was engineered via error-prone PCR, followed by site-saturation mutagenesis. The recombinant *E. coli BL21* (DE3) PPA titer reached 10.0 ± 0.4 g/L with a substrate conversion rate of 100%. Compared to the wild type, the titer was 3.0 times higher [35].

### α-ketoisocaproate

α-ketoisocaproate (KIC) is the precursor of the branched-chain amino acid leucine. α-KIC has several applications, including its use as a nitrogen-free substitute for leucine to decrease the accumulation of waste nitrogen in patients with chronic kidney and hepatic disorders. KIC can also be used as a nutraceutical, as it promotes muscle recovery after exercise-induced damage [17,36].

α-KIC is mainly chemically synthesized from isobutyl-aldehyde and acetone under harsh reaction conditions and after multiple purification steps [37]. α-KIC can also be produced via the engineering of *Corynebacterium glutamicum* biosynthesis pathways; The deletion of the *ilvE gene* in *C. glutamicum* resulted in α-KIC formation since the transamination of 2-Ketoisocaproate to L-leucine were stopped by the deletion of the responsible gene *ilvE* [17,38]. Another approach used the microbial fermentation or biotransformation to produce α-KIC via the free whole-cell biotransformation of *Rhodococcus opacus* DSM 43,250 using L-leucine as a substrate. After optimizing the conditions with response service methodology, the maximal α-KIC production reached 1.2 g/L [17,39]. A similar approach was used by Song et al. (2015) to produce α-KIC, but in this case, an *E. coli* whole-cell biocatalyst, expressing an L-AAD, EC 1.4.3.2 from *Proteus vulgaris*, was used. The membrane-bound L-AAD from *P. vulgaris* is an oxidoreductase with flavin adenine dinucleotide as a coenzyme, which catalyze the deamination of the deamination of L-leucine to KIC. The catalysis occurs via the binding of *Proteus* L-AADs to the electron transport chain to reduce O_2_ to H_2_O. After optimizing the reaction conditions and the substrate feeding strategy, the α-KIC titer reached 69.1 g/L with a substrate conversion rate of 50.3% [40]. However, the α-KIC production was low, because of feedback reactions that limited its production. In an attempt to fix the low production, Song et al. (2017) tuned the transcriptional and translational levels for L-AAD expression and optimized the plasmid origin with different copy numbers. The α-KIC production improved to 86.55 g/L with a higher leucine conversion rate of 94.25%, compared to the wild type (titer: 69.1 g/L and leucine conversion rate: 50.3%) [41]. The biocatalyst activity increased to 31.77% compared to the results previously obtained using the wild type.

### α-ketoglutaric acid

α-ketoglutaric acid (α-KG) is an important intermediate in the Krebs cycle and amino acid metabolism. The dietary supplement form of α-KG is believed to offer a variety of health benefits, including stabilized blood sugar levels during exercise and improved exercise endurance [42,43]. Industrially, α-KG is produced via chemical pathways such as the transamination of glyoxylic acid with sodium glutamate and copper as catalyst. This method has some drawbacks like the need to manage the copper catalyst and the lack of substrate selectivity as other organic acids were also being produced as byproducts [44]. Therefore, different studies have been carried out attempting to find an efficient way for the production of α-ketoglutaric acid via the use of microbial fermentation and enzymatic transformation [45].

Hossain et al. (2014a) were able to produce α-ketoglutaric acid from L-glutamic acid using a whole-cell biocatalyst. They noticed that the recombinant *E. col*i BL21(DE3) and *Bacillus subtilis* 168 could be potential biocatalysts by the overexpression of the L-AAD from *Proteus mirabilis* KCTC 2566. Using *B. subtilis* as a biocatalyst under optimized production conditions, the α-KG production reached a maximal yield of 4.65 g/L [46]. However, this process had a low substrate specificity and high α-KG degradation; therefore, later that same year, Hossain et al. (2014b) tried to solve these problems by eliminating the α-KG degradation pathway from the recombinant strain (*B. subtilis* 168). They knocked out the *sucA* gene encoding the key enzyme of the α-KG dehydrogenase which normally converts the α-KG into succinyl coenzyme A in tricarboxylic acid cycle. The deletion of *sucA* gene from *B. subtilis* 168 reduced the α-KG degradation. He also engineered the L-AAD from *P. mirabilis* KCTC 2566 through a series of error-prone PCR experiments to identify mutations at sites that influence the catalytic efficiency, followed by site-saturation mutagenesis at those sites. The combination of the deletion of α-KG degradation pathway and the engineering of L-AAD increased the α-KG titer from 4.65 g/L to 12.21 g/L [47]. However, the above whole-cell biocatalysis still exhibited some drawbacks, like the low substrate solubility of L-glutamic acid and low α-ketoglutaric acid production. To solve the issues, *P. mirabilis* L-AAD KCTC 2566 was subjected to eight rounds of error-prone PCR, followed by four rounds of gene shuffling. The evolved L-AAD, along with the optimized whole-cell biotransformation, produced a titer of 89.11 g/L, which was almost 7 times higher than what had been previously reported [48]. The integration of error-prone PCR and gene shuffling appeared to be an effective method to improve the catalytic performance of the α-KG.

### α-ketoisovaleric acid

α-ketoisovaleric acid (α-KIV) is as a precursor in leucine and valine synthesis. It also serves as an initial compound in vitamin B5 biosynthesis [49]. α-KIV is mainly synthesized via a multistep chemical method (ie the hydrolysis of azlactones and the Grignard reagents with diethyloxamates) [1,15]. These chemical processes are costly and complex which restrain the high industrial production of α-KIV.

Li et al. (2017) studied an alternative biotechnological way by expressing the L-AAD from *Proteus myxofaciens* ATCC 19,692 in *E. coli* BL21 (DE3) as a whole-cell biocatalyst system. Under the optimized conditions, the α-ketoisovaleric acid production with the wild type L-AAD was 2.014 g/L. Using the 3D structural model of L-AAD from *P. myxofaciens* and the simulation results when docking with the L-valine, key amino acid residues (N100, Q276, R316, and F318) were identified as potential target for site-saturation mutagenesis. The evolved L-AAD improved the biotransformation to 8.197 g/L after combining the mutated sites [50]. The rational molecular engineering of the L-AAD using site-saturation mutagenesis improved the efficiency of the biocatalysis.

### α-keto-γ-methylthiobutyric acid

α-keto-γ-methylthiobutyric acid is a keto acid derivative of L-methionine. Methionine is an essential amino acid in humans and poultry that is primarily obtained through dietary means. The free form of methionine can be easily degraded by the bacterial flora of the intestine, and for this reason, only a few small portions of L-methionine can be transported into the blood. The availability of the L-methionine-corresponding keto acid (α-keto-γ-methylthiobutyric acid) can be used as an alternative. α-keto-γ-methylthiobutyric acid can also be used in the poultry industry and pharmaceutical industry for the production of drugs that can treat cancer [51,52]. α-keto-γ-methylthiobutyric acid is being used in livestock feed as a methionine supplement without any reported toxicities [52].

α-keto-γ-methylthiobutyric acid is synthesized via chemical processes starting from the ethyloxylyl. Interest has increased in the production of α-keto-γ-methylthiobutyric acid from L-methionine through cell biocatalysts. García-García et al. (2008) produced α-keto-γ-methylthiobutyric acid via enzymatic synthesis by using D-amino acid oxidase (D-AAO; EC 1.4.3.3) from *Trigonopsis variabilis* CBS 409 and D-methionine as substrate. But the reaction produced also the hydrogen peroxide as a byproduct. As a result, the enzyme catalase was also needed to decompose the hydrogen peroxide to water and oxygen, which resulted in a high cost for α-keto-γ-methylthiobutyric acid [53]. An alternative process was studied to produce α-keto-γ-methylthiobutyric acid from L-methionine by the use of an *E. coli* BL21(DE3) whole-cell biocatalyst overexpressing an L-AAD from *Proteus vulgaris*. The molar conversion of L-methionine to α-keto-γ-methylthiobutyric acid reached 71.2% (mol/mol). After the directed evolution of the L-AAD from *P. vulgaris* using error-prone PCR and the screening of approximately 10^4^ mutants, two mutants were selected; one with lysine 104 substituted with arginine and the other one with alanine 337 mutated to serine. These mutations increased the molar conversion to 82.2% for the lysine to arginine substitution and to 80.8% for the alanine to serine substitution. Furthermore, these two mutations were combined, and they increased the catalytic activity and molar conversion ratio by 1.3-fold and to 91.4%, respectively, with an α-keto-γ-methyl-thiobutyric acid concentration of 63.6 g/L [54].

### Pyruvate

Pyruvate is a key intermediate in several cellular metabolic pathways. It has several applications, including its use as a nutraceutical, antioxidant, dietary supplement, and weight control supplement [55,56]. Pyruvate is produced by chemical, and biotechnological processes. The chemical process includes the dehydration and decarboxylation of tartaric acid [15]. The biotechnological processes for pyruvate production include the direct fermentation, enzymatic production, and whole-cell biocatalysis. Wang et al. (2005) metabolically engineered *Torulopsis glabrata* strain by disrupting the pyruvate decarboxylase gene responsible of the pyruvate degradation to acetaldehyde and ultimately to ethanol. The engineered strain displayed higher pyruvate accumulation and less ethanol production as byproduct [57]. Pyruvate can also be produced from fumarate through microbial cycling-imide transforming pathway [58].

Hossain et al. (2016) investigated a one-step pyruvate production from D/L-alanine using a whole-cell *E. coli* biocatalyst expressing L-AAD from *Proteus mirabilis* (PM1). The titer obtained was low, due to the substrate (alanine) and product (pyruvate) utilization by the biocatalyst. To solve that issue, the alanine uptake transporters (*cyc*A and *amaP*) and the pyruvate uptake transporter (*lldP*) were knocked out. The latter knockout increased the titer production of pyruvate from 1.14 g/L to 5.38 g/L with the mutant whole-cell biocatalyst. To improve the catalytic activity, the L-AAD (pm1) was engineered using the directed evolution method; three rounds of error-prone PCR generated the mutant pm1ep3, which showed an improved affinity and catalytic efficiency. The pyruvate production titer improved to 14.57 g/L, and the conversion ratio increased to 29.14% by use of the mutant whole-cell biocatalyst and the evolved L-AAD (pm1ep3) [59]. The combination of transporter engineering and directed evolution of the enzyme improved the pyruvate production via a whole-cell biocatalyst.

Melis et al. (2018) were able to produce 1-naphtylpyruvic, the corresponding keto acid for the synthetic amino acid L-1-naphthyalanine (L-1-Nal), using an evolved L-AAD enzyme from *Proteus myxofaciens* (PmaLAAD). Through the use of molecular docking and an evolutionary conservation analysis, they identified eight evolvable positions within PmaLAAD. The identified positions were subjected to site-saturation mutagenesis to generate libraries of variants. The best variant possessed a lower K_m_ (0.7 mM) and an approximately sevenfold higher catalytic efficiency for L-1-naphthyalanine compared to the wild-type. Hence, the semi-rational site-saturation mutagenesis of the residues in PmaLAAD, identified using a structural bioinformatic analysis, improved the catalytic efficiency of PmaLAAD toward L-1-naphythyalanine [60].

## Conclusions and future prospects

In this review, we describe the progress made on the use of whole-cell biocatalyst combined with the directed evolution to produce α-keto acids with improved activities. We discussed different case studies where the production of α-keto acids was improved using directed evolution of enzyme and microorganism-based biocatalysts. Developing the whole-cell biocatalyst with evolvable properties could turn out being an attractive way to produce keto acids. The simplicity of its reaction mixture and the inexpensive substrate could provide an economical and environmentally friendly way to produce the α-keto acids comparing to the chemical method which is costly and uses harsh chemicals [13,34,35,46–48,54,59,61].

Finally, progress in metabolic engineering of whole-cell biocatalysts is encouraging the use of biocatalysts to a whole new level. Integration of enzyme engineering with the modification of recombinant strain mechanism pathways, have shown an ability to improve the overall performance of biocatalytic processes. Nevertheless, some work still needs to be addressed like a better understanding of enzyme structure, an efficient quality library screening, and selection, to make the whole-cell biocatalysts up to the point of competing with chemical synthesis for the production of α-keto acids. But, the emerging researches in protein chemistry, computational design, directed evolution and the development of new tools for selection and screening promise the future use of biocatalysts for the production of α-keto acids on a large scale.
